# Massive pulmonary embolism presenting with hemoptysis and S1Q3T3 ECG findings

**DOI:** 10.1186/s12872-021-02035-0

**Published:** 2021-05-01

**Authors:** Mehmet Sami Islamoglu, Mehmet Dokur, Emrah Ozdemir, Omer Faruk Unal

**Affiliations:** 1 Department of Internal Medicine, Biruni University of Medical Faculty, Istanbul, Turkey; 2 Department of Emergency Medicine, Biruni University of Medical Faculty, Istanbul, Turkey; 3 Department of Cardiology, Biruni University of Medical Faculty, Istanbul, Turkey; 4 Department of Radiodiagnostic, Biruni University of Medical Faculty, Istanbul, Turkey

**Keywords:** Massive pulmonary embolism, Typical electrocardiographic findings, S1Q3T3, Hemoptysis

## Abstract

**Background:**

Venous thromboembolism clinically presenting with a deep vein thrombosis or pulmonary embolism is among the most commonly seen cardiovascular syndromes. The aim of this case presentation is to emphasise the typical electrocardiographic findings that are detected with massive pulmonary embolism along with the electrocardiographic S1Q3 and S1Q3T3 accompanied by T negativity at the D3 derivation based on prevalent T negativity.

**Case presentation:**

We present the case of an adult male who presented with a massive pulmonary embolism that was associated with tachycardia, haemoptysis and typical S1Q3T3 electrocardiographic findings. Tomographic findings showed filling defects in the two main pulmonary artery lumens, which were found to be compatible with a massive embolism. Intravenous heparin was injected (5000 IU), and low molecule weight heparin (LMWH) treatment was initiated. After two days of observation and treatment in the coronary intensive care unit, the patient was discharged for outpatient care.

**Discussion:**

Massive pulmonary embolism is an urgent life-threatening clinical situation that is frequently confused with acute ST elevation myocardial infarction. The definitive diagnosis of massive pulmonary embolism was made with a computed tomography pulmonary angiogram. Electrocardiographic findings and hypoxic hypercarbia in the blood gas analysis are typical. Early diagnosis with laboratory and imaging investigations is vital in the treatment and prognosis of pulmonary embolism.

**Conclusions:**

Ventricular overload signs accompanied by ST segment elevation in electrocardiography and S1Q3 and prevalent T negativity are crucial features in terms of distinguishing between pulmonary embolism and myocardial infarction and selecting effective treatments for patients admitted to the emergency department.

## Background

Venous thromboembolism clinically presenting with a deep vein thrombosis or pulmonary embolism (PE) is among the most commonly seen cardiovascular syndromes [1]. The incidence of PE is 39–115 per 100,000, and deep vein thrombosis is 53–162 per 100,000 [2, 3]. In 30 to 50% of PE cases, deep vein thrombosis was also observed. In the case of proximal deep vein thrombosis, PE has a progressive and increased risk of early death. In the United States of America, there are around 300,000 PE-related deaths per year [2]. In recent years, effective use of treatments and effective implementation of standard guidelines have had positive effects on PE prognosis [4]. Although clinical findings and symptoms are not very specific in PE cases, the majority of cases present with breathing difficulty, chest pain, syncope and haemoptysis. Haemodynamic instability is rarely observed and is generally seen in central or massive embolism cases [4]. In suspected high-risk PE, as indicated by the presence of haemodynamic instability, bedside echocardiography or emergency computed tomography pulmonary angiogram (CTPA) are recommended for diagnosis. Plasma D-dimer measurement is recommended in emergency department patients with low or intermediate clinical probability. It is recommended to reject the diagnosis of PE if the CTPA is normal in a patient with low or intermediate clinical probability or who is unlikely to have a PE [4]. Electrocardiography (ECG) is a relatively cheap, easily accessible and non-invasive tool, and there are many studies that have determined its prognostic value. The majority of PE cases show sinus tachycardia on ECG; T negativity in V1 and ST elevation in aVR are also prominent [5]. This case presented to the hospital with haemoptysis, chest pain and difficulty breathing. Clinically, massive PE was suspected along with electrocardiographic S1Q3 and S1Q3T3 features accompanied by T negativity at the D3 derivation based on prevalent T negativity. There was also right bundle branch block (RBBB), sinus tachycardia and ST elevation in aVR.

In this report, we present the case of an adult male with typical (S1Q3T3) electrocardiographic findings who we diagnosed with massive PE and also provide context in light of the current literature.

## Case presentation

**Fig. 1:**
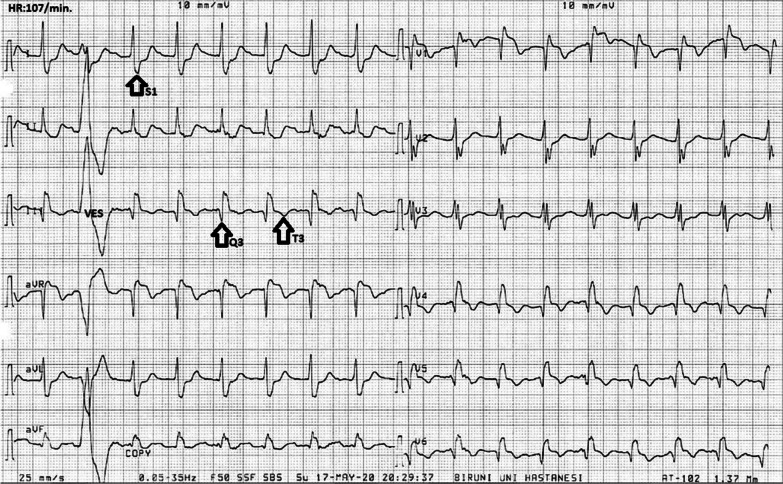
ECG Right Bundle Branch Block, deep S wave in S1 I. Derivation, Q wave and T negativity (S1Q3T3) in III. derivation, ST elevation in the AvR and T negativity in II-III and aVF

**Fig. 2:**
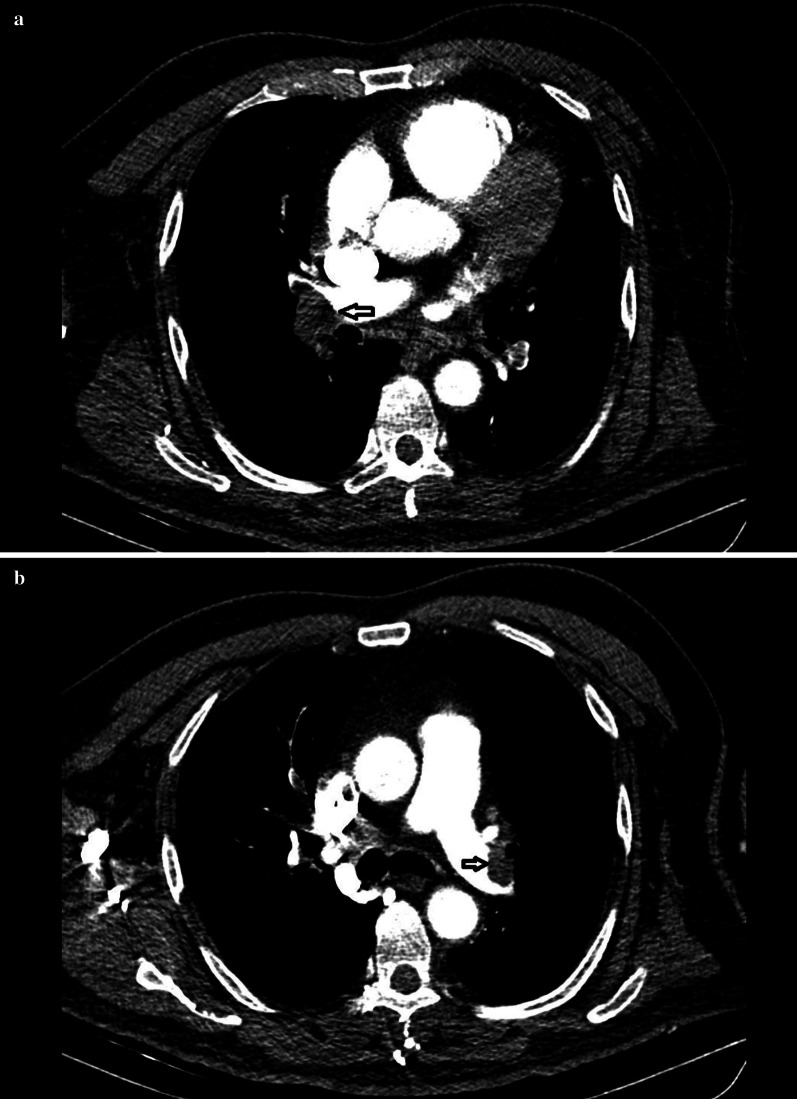
Massive pulmonary artery embolism appearance on thorax computerized tomography angiography (CTPA). **a** Filling defect inside right pulmonary artery embolism on CTPA. **b** Filling defect inside left pulmonary artery embolism on CTPA

A 61-year-old male patient was admitted to our hospital with chest pain, difficulty breathing, haemoptysis and general discomfort. His body mass index was 35, which is consistent with obesity, and he also had hypertension based on anamnesis. The patient was conscious, but there was a lack of cooperation and orientation. The Glasgow Coma Scale was 13 (eye = 6, motor = 4, verbal = 3), S1 (+) and S2 (+). His heart rate was 120/min, body temperature was 37 °C, oxygen saturation was 89%, systolic blood pressure was 90 mm/hg and diastolic blood pressure was 60mm/Hg. The ECG results diagnosed RBBB and deep S wave (S1) in I; derivation, Q wave and T negativity (S1Q3T3) in III; derivation, ST elevation in aVR and T negativity in II–III and aVF (Fig. 1). After the patient was diagnosed with hypotension, chest pain and ST elevation in aVR, coronary angiography for myocardial infarction displayed normal results, and medical follow-up was suggested for the plaques. In order to isolate the right myocardium, another ECG was performed, and it displayed normal results. In addition to a positive Homan’s sign, there was swelling and erythema of the left leg. Doppler ultrasound displayed hypoechoic thrombus material with echogenic focus points inside the lumen throughout the left superficial femoral vein proximal to the distal femur level. According to the Geneva scoring chart included in the 2019 European Society of Cardiology Guidelines for the diagnosis and management of acute pulmonary embolism, there was a high clinical probability of PE, with 14 points, due to heart rate higher than 95/min (5 points), haemoptysis (2 points), unilateral oedema (4 points) and unilateral lower leg pain (3 points). The Wells score also indicated a high probability of PE, with 8.5 points, due to heart rate higher than 100/min (1.5 points), haemoptysis (1 points), clinical signs and symptoms of deep venous thrombosis (3 points) and alternative diagnosis less likely than PE (3 points). CTPA was conducted to confirm the diagnosis. The CTPA results reinforced the probability of a massive PE and displayed filling defects inside the two main pulmonary artery lumens; the right was more apparent. Another filling defect was observed in the pulmonary artery lumen leading to the upper lobe of the left lung, again reinforcing the probability of PE (Fig. 2a, b). Laboratory tests indicated hypoxemic hypocarbia and acidosis in the blood gas analysis (pH:7.16, pCO2:35 mmHg, PO2:83 mmHg, SO2:92%, lactate:11.2 mmol/L and anion gap: 24 mmol/L). The following parameters further favoured the diagnosis of PE: urea:48 mg/dL, creatinine:1.5 mg/dL, AST:48 U/L, ALT:39 U/L, WBC:15.7 K/µL, Hb:16 g/dL, PLT:209 K/µL, CRP:15 mg/L, troponin I:41 pg/ml and D-dimer:4529 ng/mL. Due to the hypotensive status of the patient, continuous fluid infusion was administered upon admittance to the emergency department. Continuous oxygen support (4 L per minute) was also provided. Intravenous heparin was injected (5000 IU). During the observation period, further laboratory investigations revealed high CRP:65 mg/L and troponin I:2191 g/mL. LMWH treatment (2 × 6000IU) was started. After two days of observation and treatment in the coronary intensive care unit, the patient was discharged for outpatient care.

## Discussion and conclusions

The ECG results in this case diagnosed RBBB and deep S wave (S1) in I; derivation, Q wave (Q3) and T negativity (T3) in III; derivation, named as (S1Q3T3), ST elevation in aVR and T negativity in II–III and aVF. Because S1Q3T3 indicated severe disease and was responsible for the patient’s state of shock, this finding makes this case special.

Massive PE is an urgent life-threatening clinical situation that is frequently confused with ST elevation acute MI and other diseases, such as acute heart failure, pneumonia, asthma, pericarditis, pleuritis and pneumothorax. Clinical findings, laboratory tests and radiologic investigations are useful in the differential diagnosis of PE [4]. Old age, prolonged immobility, surgery, fracture, oral contraceptive use, hormone replacement therapy, pregnancy, puerperium, cancer and antiphospholipid syndrome are risk factors for venous thromboembolism [6]. Besides activated protein C resistance (factor V Leiden), prothrombin G20210A deficiency, hyperhomocysteinemia, protein C, protein S, antithrombin III and thrombomodulin defects are the most prominent hereditary thrombotic disorders [7]. The presence of several mutations at the same time can significantly increase susceptibility to the disease. Environmental factors can interfere with one or more genetic variants to increase the risk even further [6].

In the evaluation of PE with ECG, T negativity in V1–V4, which indicates right ventricular overload, QR, S1Q3T3 in V1 and RBBB are observed in severe cases, whereas only sinus tachycardia is observed in mild cases [8]. Atrial fibrillation is the most common among atrial arrhythmias in PE. In 54 studies conducted by Shopp et al. in 8209 patients, the rates of ECG findings were as follows: tachycardia (38%), T inversion in V1 (38%), ST elevation in aVR (36%), S1 (33%), Q3 (32%), T3 inversion (30%) and S1Q3T3 (24%) [9]. In our case, there was S1Q3T3, ST elevation in aVR, T negativity and RBBB in II–III/aVF, ST elevation in V1 and aVF and prevalent T negativity in V1–V6. In a study conducted by Kukla et al. in 255 patients, patients with higher levels of troponin had S1Q3T3, T negativity in V2–V4, V4–V6 ST segment depression, V1–V3 ST segment elevation and QR and prevalent T negativity in V1 when compared to patients with normal troponin levels [9]. In the same study, patients with S1Q3T3 and other ECG findings displayed higher risks of acute PE-related death in hospital. In our case, the troponin I level was high in harmony with the ECG findings. Initial and follow-up troponin I levels were 41 pg/mL and 2191 pg/mL, respectively. In a meta-analysis of 10 studies that included 3007 patients, heart rates were found to be higher than 100/min. S1Q3T3, complete RBBB, inverted T waves in V1–V4, ST elevation in aVR and atrial fibrillation were found to be associated with circulatory collapse and shock [8]. In our study, the patient with tachycardia and typical ECG findings presented in state of shock which is similar to the meta-analysis. In a study conducted by Zhan et al. in 20 patients with haemodynamic instability, S1Q3 and abnormal QRS in V1 were detected in 90% of the patients. In this study, it was hypothesized that S1Q3 is important in terms of right ventricular overload and risk evaluation and that T negativity in the III derivation develops after ischemia on the right ventricle, as is the case in other derivations [10].

In conclusion, sudden death is observed in the majority of cases of massive PE, which is considered among the acute cardiovascular syndromes, and diagnosis can only be made postmortem [11]. Early diagnosis using echocardiography or CTPA is strongly suggested in patients with haemodynamic instability. PE might be isolated in patients who do not display instability. Thus, they can be evaluated in the low-risk group using non-invasive investigations [12]. The presence of specific ECG features is one way to distinguish PE from myocardial infarction. This patient survived due to the correct choice of tests, early diagnosis and appropriate treatment. For patients admitted to the emergency service with massive PE findings, ventricular overload signs accompanied by ST segment elevation and S1Q3 and prevalent T negativity are crucial in terms of distinguishing PE from myocardial infarction and selecting effective treatments.

## Data Availability

Data sharing is not applicable to this article as no datasets were generated or analysed.

## Abbreviations

CTPA: Computerized Tomography Pulmonary Angiogram
ECG: Electrocardiography
PE: Pulmonary embolism
MI: Miyocardial Infarction
RBBB: Right Bundle Branch Block
ESC: European Society of Cardiology
BMI: Body Mass Index
GCS: Glasgow Coma Scale
